# In-vivo durability of a fluoride-releasing sealant (OpalSeal) for protection against white-spot lesion formation in orthodontic patients

**DOI:** 10.1186/s13005-015-0069-6

**Published:** 2015-04-15

**Authors:** Michael Knösel, David Ellenberger, Yvonne Göldner, Paulo Sandoval, Dirk Wiechmann

**Affiliations:** Department of Orthodontics, University Medical Center Göttingen (UMG), 37099 Göttingen, Germany; Department of Medical Statistics, University Medical Center Göttingen (UMG), 37099 Göttingen, Germany; Private Practice, Hannover, Germany; Department of Orthodontics, Universidad de la Frontera (UFRO), Temuco, Chile; Orthodontic Practice, Lindenstrasse 44, 49152 Bad, Essen, Germany; Department of Orthodontics, Hannover Medical School (MHH), 30625 Hannover, Germany

**Keywords:** Orthodontic sealant, Durability, OpalSeal, White-spot lesions, In-vivo

## Abstract

**Background:**

Sealant application during fixed appliances orthodontic treatment for enamel protection is common, however, reliable data on its durability in vivo are rare.

**Objective:**

This study aims at assessing the durability of a sealant (OpalSeal, Ultradent) for protection against white-spot lesion formation in orthodontic patients over 26 weeks in vivo, taking into account the provision or absence of an adequate oral hygiene. We tested the null hypothesis of (1) no significant abatement of the sealant after 26 weeks in fixed orthodontic treatment compared to baseline, and (2) no significant influence of the factor of brushing and oral hygiene (as screened by approximal plaque index, API) on the abatement of the sealant.

**Methods:**

Integrity and abatement of OpalSeal applicated directly following bracketing was assessed in thirty-six consecutive patients (n_teeth_ = 796) undergoing orthodontic treatment with fixed appliances (male/female12/24; mean age/SD 14.4/1.33 Y). Assessment of the fluorescing sealant preservation was by a black-light lamp, using a classification that was concepted in analogy to the ARI index: (3, sealant completely preserved; 2= > 50% preserved; 1 = <50%; 0 = no sealant observable) immediately following application (Baseline, T0), after 2 (T1), 8 (T2), 14 (T3), 20 (T4) and 26 weeks (T5). API was assessed at T0 and T1. Statistical analysis was by non-parametric repeated measures ANOVA (α = 5%, power >80%).

**Results:**

At baseline, 43.4% of teeth had a positive API. Oral hygiene deteriorated after bracketing (T1, 53%) significantly. Null hypothesis (1) was rejected, while (2) was accepted: Mean values of both the well brushed and non-brushed anterior teeth undercut the score “1” at T3 (week 14). Despite a slightly better preservation of the sealer before and after T3 in not-sufficiently brushed (API-positive) teeth, this finding was statistically not significant.

**Conclusion:**

One single application of OpalSeal is unlikely to last throughout the entire fixed appliance treatment stage. On average, re-application of the sealant can be expected to be necessary after 3.5 months (week 14) in treatment.

## Introduction

Prevention of white-spot lesions (WSL) during fixed appliances orthodontic treatment is still a challenge in today’s orthodontic treatment: There is evidence that neglecting oral hygiene during orthodontic treatment with fixed appliances can cause WSL formation within weeks [1-4]. Other than mechanical plaque removal by tooth brushing, local fluoridation by dentifrices and mouth rinses, or the use of fluoride-releasing bonding materials, major preventive strategies for a prevention of enamel demineralization during fixed orthodontic treatment focus on the application of fluoride-releasing sealants [5,6].

Sealant application for enamel protection is common in fixed appliances orthodontic treatment patients, however, reliable data on its durability in vivo are rare [7]. Tüfekçi et al. investigated the preservation of a sealant on extracted premolars 67 ± 28 days following bracket bonding and sealant application in vivo, and found that layers of OpalSeal (Ultradent) remained on an average of 50% at the time of assessment, and found no correlation between sealant residues and the variation of time the teeth were in the mouth [7]. However, it is conceivable that the factors of oral hygiene and abrasion caused by mechanical tooth brushing, as well as acidic or mechanical assaults during consumption of food and beverages may have an impact on the sealant condition and durability in vivo: Varnish layers may be reduced in thickness and extension by daily mechanical wear. However, whilst there have been studies on reduction of WSL occurrence following fluoride-releasing sealant application, there is a lack of studies concerning an vivo-screening of the integrity or abatement of sealants, in interference with oral hygiene habits and observation time.

### Study aims

This study aims at assessing the durability of a sealant (OpalSeal, Ultradent Products, South Jordan, Utah) for protection against white-spot lesion formation in orthodontic patients over more than six months (26 weeks) in vivo, taking into account the provision or absence of an adequate oral hygiene.

We tested the null hypotheses of (1) no significant abatement of the sealant (as screened by a score from 0–3) after 26 weeks in fixed orthodontic treatment compared to baseline, and (2) no significant influence of the factor of oral hygiene (as screened by approximal plaque index, API [8]) on the abatement of the sealant.

## Subjects and Methods

Thirty-six consecutive patients undergoing orthodontic treatment with fixed appliances (male/female 12/24; age 12–17 years; mean age 14.44 Y; SD 1.33) were consecutively recruited at an orthodontic practice in Hannover, Germany, between Nov 1st, 2011 and April 30, 2012. Subjects were included upon meeting the following inclusion criteria:

- upcoming indirect Damon-3 (Ormco, Orange, CA, USA) bracket placement of least of sixteen teeth,
- application of a sealant (OpalSeal, Ultradent Products, South Jordan, Utah) on that same appointment, and
- having given consent for participation and accepting follow-up assessments during recall visits.

Subject were excluded upon refusal of sealant application, or less than sixteen teeth bracketed, or if they disagreed to participate. Other than exclusion of subjects, single teeth of included trial subjects were not assessed by this study in case they were not bracketed on the same appointment, or in case they were subject to upcoming extraction. Of 864 potentially eligible teeth, a number of 796 trial teeth was included (drop-out: n = 68 teeth).

Standardized indirect bracket placement using a dry-field system for isolation was performed prior to sealant application, in order to allow for a removal of excessive adhesives without setting damages to sealant layers. Following cleaning of tooth surfaces with fluoride-free pumice, adhesive and sealant application routine was carried out following manufacturer’s instructions and included a 15 s interval of etching with 37 % phosphoric acid of the complete labial enamel surface, followed by indirect bonding using chemically-cured Monolok2 composite adhesive system (Rocky Mountain Orthodontics, Denver, Colo, USA). Adhesive residues have been removed prior to sealant application. According to the manufacturer’s instructions, OpalSeal was gently air-dried following application, prior to light-curing for 20s per tooth (Bluephase C8, 800 mW/cm2, IvoclarVivadent, Schaan, Liechtenstein).

### Ethical approval

The study was performed in extension of an earlier positively voted study protocol (# 4/8/09). All procedures used in this prospective observational study had been presented to the ethics committee of the University of Göttingen, Germany, earlier. There were no objections against publication. The patients and their guardians gave informed consent for taking part in the study.

### Parameter 1: Screening of oral hygiene

The approximal plaque index (API) has been introduced in dentistry for a quick assessment of oral hygiene status [8]. Although being based on more or less subjective decisions that are made chair-side, API assessments have been established as a basic clinical methodology used in research on the subject of cariology and periodontology [9]. Oral hygiene status was screened using the API for each bracketed tooth, as a yes/no decision (results given in % of teeth with plaque) prior to bracket placement and sealant application at T0, and after 14 days in treatment (T1). All patients received identical, standardized instructions on both tooth- and inter-bracket brushing during orthodontic treatment with fixed appliances, and were advised to do so three times daily, using typical commercially available 1,400-1,450 ppm fluoridated dentifrices. They were provided with the same type of tooth brushes with medium filaments, and interdental brushes (TePe, Malmö, Sweden).

### Parameter 2: Scoring of sealant layer integrity

**Figure 1:**
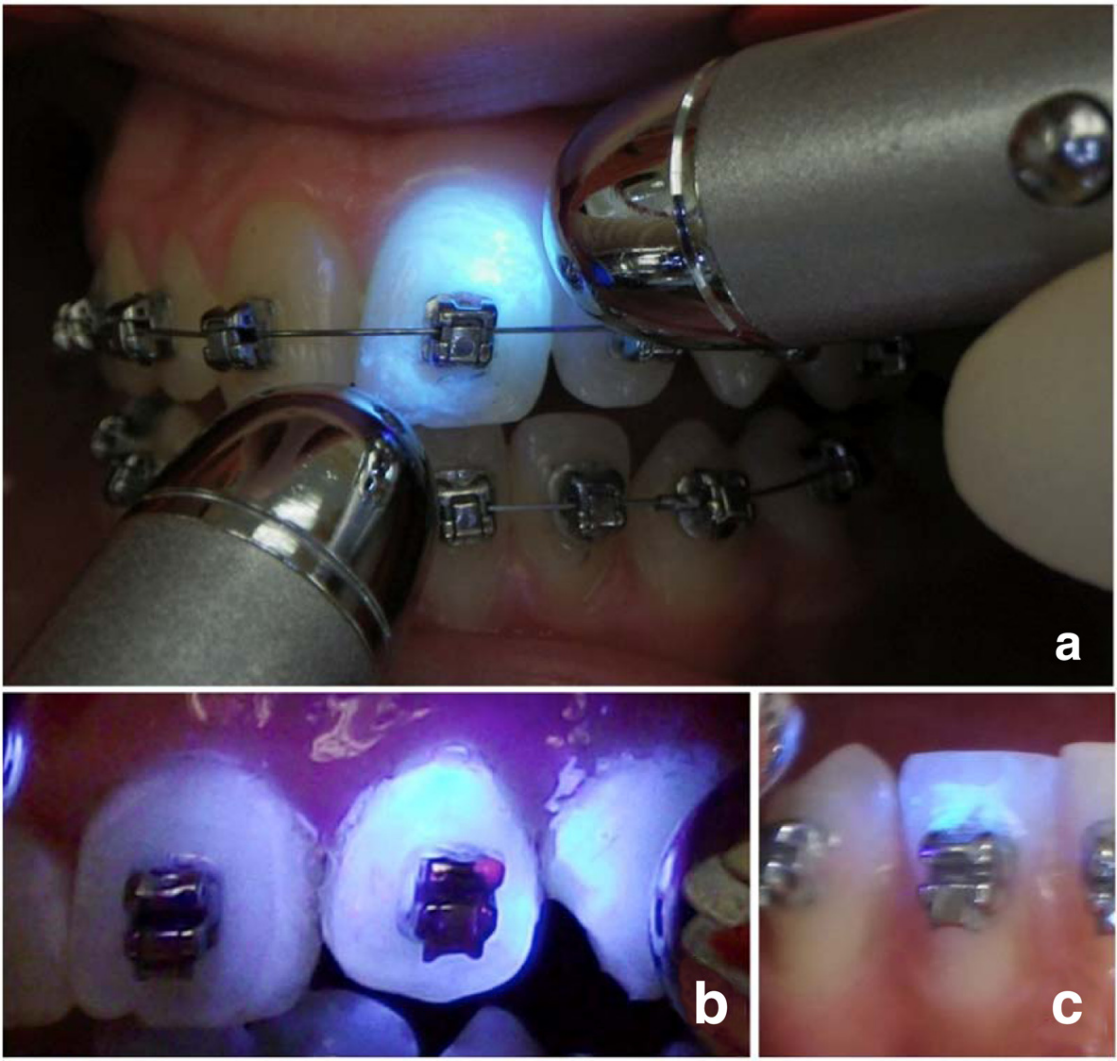
**a** Assessment of sealant integrity was done by black-light illumination, using the fluorescent properties of the OpalSeal. **b** and **c** give examples of sealant scores 3 (sealant undamaged/completely preserved), and 1 (<=50% of sealant left).

Integrity and condition of the OpalSeal-layer was assessed using a black-light UV lamp provided by the manufacturer for screening purposes of the fluorescing sealant. Similar to previous trials [4], assessments were done chair-side by a clinician who was blinded to the patient’s trial time frame, while notes were made by a study nurse: Immediately after bonding and sealant application (Baseline, T0), after 14 days (T1), 8 weeks (T2), 14 weeks (T3), 20 weeks (T4) and 26 weeks (T5). Abatement of the varnish was parameterized using a classification from 0 to 3 that was concepted in analogy to the adhesive remnant index (ARI, [10]): (3 = sealant undamaged/completely preserved, 2 > =50% preserved, 1 < =50%, 0 = no sealant observable to the naked eye), assessed for every bracketed tooth (max. 24 per patient, Figure 1a, b and c).

### Statistical analysis

The factor of ‘oral hygiene’ as assessed by API scores at Baseline (T0) and two weeks following bracketing (T1) was tested for potential changes (increases in API score = deterioration of oral hygiene) using a t-test for dependent samples. The status of the durability of the OpalSeal-layer as well as potential impacts and interactions of the initial API (T0), trial time elapse (T1-T5), tooth type (#1-#6; 1, central -; 2, lateral incisor; 3, canine; 4, first-; 5, second premolar; 6, first molar) and jaw (maxilla, mandible) were tested by non-parametric, repeated measures ANOVA, with the OpalSeal-Score as dependent variable. Correlated measurements within one patient as well as over time for each tooth were modeled by a random factor ‘subject’ along with a random factor ‘tooth’ yielding a nested compound symmetry structure. In the case of significant interactions between the experimental factors, the data were split and further analyzed in subgroups. The significance level was set to 5%. Sample size calculation according to O’Brien-Castelloe yielded a power in excess of 80% for an inclusion of 36 subjects/796 teeth. All analyses were performed using SAS 9.3 (SAS Institute, Cary, NC, USA) and Statistica 10 (StatSoft (Europe) GmbH, Hamburg, Germany).

## Results

At baseline, 43.4% of teeth had a positive API (SD: 20.5%). Oral hygiene deteriorated after bracketing (T1, 53%, SD: 22.0%) significantly: The T-test for dependent samples API (complete, %) T0 vs T1 yielded p = 0.01.

### Effect of oral hygiene (API) on sealant abatement

**Figure 2:**
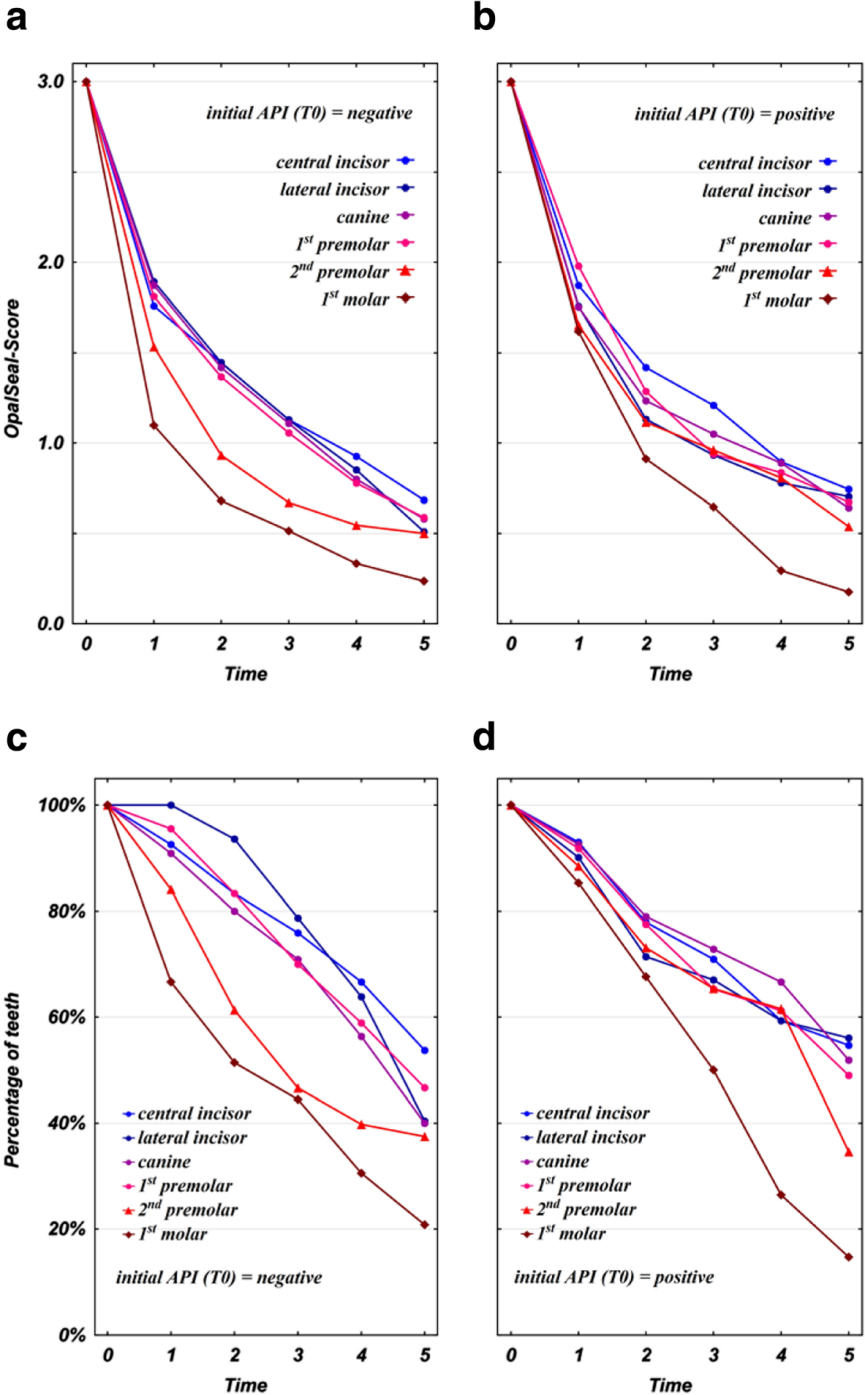
**a**, **b** Sealant layer abatement by mean OpalSeal-scores in sub-groups with positive or negative API scores indicate a significant increase in abatement from front teeth to posterior teeth (see also Table 1). On average, well brushed (left) and non-brushed (right) anterior teeth undercut the 50% sealant presevation (score “1) at T3 (week 14). The slightly better sealant preservation before and after T3 in API-positive teeth was globally not significant. On average, re-application of sealant can therefore be expected to be necessary after 3.5 months in active treatment. **c**, **d** Percentages of teeth with sealant scores higher than 0. At T5, approximately 50% of front teeth #1-#4 with positive API had at T5 a sealant score higher than 0, while in the case of well brushed teeth percentages were slightly lower. See also Table 3 for details.

At T1, we found that teeth with positive API scores showed no significant differences in terms of sealant layer preservation, in contrast to teeth with negative API scores (Table 1). Generally spoken, there was an increase in the abatement of the sealant from front teeth 1–4 to posterior teeth #5 or #6, which is globally significant (Table 2) and was found to be more rapid in well brushed lower posterior teeth (teeth #5 and #6 with negative API). (Table 1, Figures 2a and b). Mean values of both the well brushed and non-brushed anterior teeth undercut the score “1” (<50% sealer left) at T3 (week 14) (Figure 2a and b). Percentages of teeth with a score higher than 0 are depicted by Table 3 and Figure 2c and d. Despite the overall slightly better preservation of the sealant before and after T3 in not-sufficiently brushed (API positive) teeth compared to API-negative teeth, this finding was statistically not significant (Table 2). That is, considering the total trial time, the factor oral hygiene itself has no global significant effect on the abatement of the sealant. See (Figure 2a and b) for a visualisation of this effect.

**Table 1:** **As anterior teeth #1-#4 were found to be homogeneous in terms of abatement of the sealant score, pair-wise comparisons between this group of teeth with teeth #5 and #6 were implemented**

**Time**	**API (T0)**	**Tooth groups compared**	**Opalseal score difference**	**Standard error**	**p-value**	
1	positive	1-4	5	0.24	0.16	0.20
1	positive	1-4	6	0.26	0.12	0.07
1	positive	5	6	0.03	0.19	0.94
1	negative	1-4	5	0.21	0.08	0.02
1	negative	1-4	6	0.69	0.09	<.0001
1	negative	5	6	0.49	0.10	<.0001
2	positive	1-4	5	0.26	0.16	0.08
2	positive	1-4	6	0.41	0.12	0.001
2	positive	5	6	0.15	0.19	0.57
2	negative	1-4	5	0.39	0.08	<.0001
2	negative	1-4	6	0.69	0.08	<.0001
2	negative	5	6	0.31	0.10	0.005
3	positive	1-4	5	0.20	0.16	0.15
3	positive	1-4	6	0.45	0.12	0.0002
3	positive	5	6	0.25	0.19	0.25
3	negative	1-4	5	0.33	0.08	<.0001
3	negative	1-4	6	0.54	0.09	<.0001
3	negative	5	6	0.21	0.10	0.07
4	positive	1-4	5	0.22	0.16	0.17
4	positive	1-4	6	0.61	0.12	<.0001
4	positive	5	6	0.39	0.19	0.03
4	negative	1-4	5	0.21	0.08	0.003
4	negative	1-4	6	0.46	0.09	<.0001
4	negative	5	6	0.26	0.10	0.02
5	positive	1-4	5	0.30	0.16	0.04
5	positive	1-4	6	0.58	0.12	<.0001
5	positive	5	6	0.27	0.19	0.13
5	negative	1-4	5	0.00	0.08	0.79
5	negative	1-4	6	0.32	0.09	<.0001
5	negative	5	6	0.32	0.10	0.001

Especially during the first weeks in treatment, sealant preservation was better in API-negative teeth, although this finding was globally not significant when considering all time points (see also Table 3).

**Table 2:** Factors and interactions that have a potential impact on sealant durability scores

**Effect**	**ANOVA**
	**p-Value**
Jaw (Maxilla, Mandible)	<.0001
Tooth type (#1,#2,#3,#4,#5,#6)	<.0001
Jaw * Tooth type	0.01
Time (T 1,2,3,4,5)	<.0001
Jaw * Time	0.45
Tooth type * Time	0.69
Jaw * Tooth type * Time	0.83
Oral hygiene by initial API (0)	0.54
Jaw * Oral hygiene	0.24
Tooth type * Oral hygiene	0.73
Jaw * Tooth type * Oral hygiene	0.26
Time * Oral hygiene	0.10
Jaw * Time * Oral hygiene	0.08
Tooth type * Time * Oral hygiene	0.0002
Jaw * Tooth type * Time * Oral hygiene	0.83

The explained variance by within-subject measurements was found to be crucial with R^2^ = 0.34 (p < .0001) for the random factor ‘subject’ and R^2^ = 0.27 (p < .0001) for the random factor ‘tooth’.

**Table 3:** **Sealant abatement: Percentages of teeth with sealant scores higher than 0 in sub-groups with adequate or inadequate oral hygiene (negative or positive API scores)**

***Tooth type #***	***Time***	**Teeth with sealant score 1 or higher (%)**	
		**Negative API**	**Positive API**
1	0	100	100
1	1	92.59	93.02
1	2	83.33	77.91
1	3	75.93	70.93
1	4	66.67	59.30
1	5	53.70	54.65
2	0	100	100
2	1	100	90.11
2	2	93.62	71.43
2	3	78.72	67.03
2	4	63.83	59.34
2	5	40.43	56.04
3	0	100	100
3	1	90.91	91.84
3	2	80.00	77.55
3	3	70.91	65.31
3	4	56.36	61.23
3	5	40.00	48.98
4	0	100	100
4	1	95.56	92.59
4	2	83.33	79.01
4	3	70.00	72.84
4	4	58.89	66.67
4	5	46.67	51.85
5	0	100	100
5	1	84.09	88.46
5	2	61.36	73.08
5	3	46.59	65.38
5	4	39.77	61.54
5	5	37.50	34.62
6	0	100	100
6	1	66.67	85.29
6	2	51.39	67.65
6	3	44.44	50.00
6	4	30.56	26.47
6	5	20.83	14.71

### Maxilla vs. Mandible

**Figure 3:**
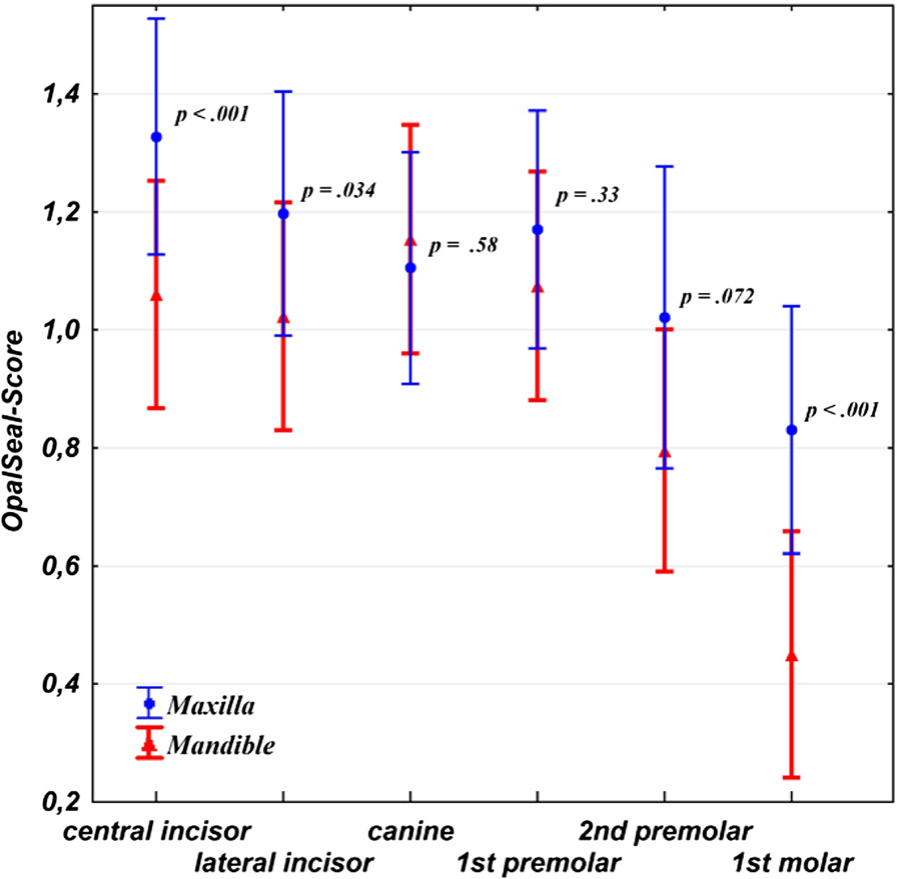
Pairwise comparisons of time-averaged mean OpalSeal-scores (with 95% confidence intervals) stratified by tooth type (#1-6) and jaw (maxilla, mandible). Abatement of sealant layer was significantly increased in the mandibular teeth #1, #2, #6 (p < 0.001; 0.03; <.0001) when compared to the maxillary equivalent.

Pairwise comparisons indicate a more pronounced abatement of the sealant in the mandible than in the maxilla at T1, and it was significantly increased in mandibular teeth #1, #2, and #6 when compared to the maxillary equivalent (Figure 3).

## Discussion

An inhibition of enamel demineralization during orthodontic fixed treatment can be achieved by the application of fluoride-releasing sealants [11,12], however, the efficacy of those sealants also depends on their integrity or durability [7]. It is a popular fallacy to assume that one sealant application at the start of fixed appliances orthodontic treatment will suffice for enamel protection throughout the entire fixed treatment stage, without a renewal [13,14]. In-vivo research yielded evidence that sealants offer some protection and are suitable for reducing frequencies of new WSL [12], but do not offer outright protection from WSL formation for the full duration of treatment [7]. Diligence during application and frequencies of re-application may be relevant in terms of sealant durability, as may be the presence of different levels of oral hygiene and intensities of tooth- and inter-bracket brushing as a factor that is potentially causing sealant abrasion. In-vivo data on the durability of those sealants are scarce: A recent in vivo report on sealant preservation in premolars extracted following 67 ± 28 days found that an average of 50% of OpalSeal was left on the teeth [7]. Moreover, the authors reported the reduction of WSL frequencies in OpalSeal-treated teeth as being small, but not significant in comparison to a non-fluoride releasing bonding following 90 or more days; a beneficial effect in terms of a significant reduction of WSLs was only seen in teeth assessed within the first 3 months. They concluded that this result may have been due to an abatement of the sealant, indicating the necessity for multiple applications [7]. However, there is no clear guideline for the handling of sealant re-application based on the available evidence, particularly as it is not yet known how protective effects and durability of the fluoride-releasing sealants interact with additional etching intervals that may be necessary to achieve sealant retention after months in treatment: It is a fact that additional, surplus etching of enamel surfaces not covered by bracket bases may trigger WSL formation itself [15]. The data presented here add some evidence to the unanswered questions of recommended time intervals for a renewal of the sealant, and, second, the unclear role of individual intensities of oral hygiene and mechanical tooth brushing abrasion on sealant durability: The null-hypothesis (1) of no significant global abatement of the sealant after 26 weeks in treatment was rejected, while the null hypothesis (2) was accepted, as we did not find a significant global effect of the factor of oral hygiene on the abatement of the sealant. Nonetheless, there was an increase in the abatement of the sealant from front teeth 1–4 to posterior teeth #5 or #6, which is significant (Table 1) and was found to be more rapid in well brushed lower posterior teeth (teeth #5 and #6 with negative API; Figure 2a). One possible interpretation might be that sealant layers may be literally wiped off by tooth brushing, and it may be concluded that sealants offer a slightly better protection of those teeth that are not sufficiently brushed. Another possible explanation would be that this abatement in mandibular posterior teeth may also be attributed to enhanced attrition by chewing activities. This would explain reductions of varnish layers in the upper enamel third of lower first molars and premolars, as those areas are more exposed to daily mechanical wear during consumption of food.

Mean sealant scores indicate that in both the well brushed and non-brushed teeth anterior teeth undercut the score “1” (less than 50% sealer left) at T3 (week 14), while there seems overall a slightly better preservation of the sealer before and after T3 in positive API-teeth, especially in the case of second premolars (Figure 3c). However, globally, these slightly better results are not significantly improved compared to the non-brushed teeth (Table 2). Re-application can therefore be expected to be necessary from week 14 in the average case treated with orthodontic fixed appliances. This result is in agreement with Tüfekçi et al. who reported a preservation of 50% of the same sealant following ninety days [7].

### Sealant re-application: Time intervals and mode

According to the manufacturer, re-application of OpalSeal implies prior etching for 15-30s, and subsequent light-curing for 10 s. Previous research hints at a potential iatrogenic triggering of WSL especially in those cases with surplus orthodontic etching prior to bracket bonding, especially when those areas are subsequently neither covered by bracket bases nor by bonding material [15]: This has been shown to have a significant deteriorating effect on WSL formation. Subsequent covering of those etched areas by a sealant prevents WSL formation, however, the results of the current study show that the sealant abates on average in about 3.5 months, and it is unclear whether there is an increased susceptibility to WSL after disappearance of the sealant. According to previous reports, teeth are especially susceptible to decalcification during the first six months in fixed appliances orthodontic treatment [4]. If this was due to some type of intra-oral customization to the fixed orthodontic appliances, simple re-application of sealants without repeating the step of etching may also be viable.

### Advantages and drawbacks of the study design

Integrity and condition of the OpalSeal layers was assessed chair-side by a clinician who was blinded to the patient’s trial time schedule, while notes were made by a study nurse, similar to previous studies on the topic of WSL formation in orthodontic patients [4]. The assessment of photos would not have been feasible here, as every single tooth would have to be photographed while being illuminated by the black-light lamp. However, as an advantage, the trial sample size and numbers of performed assessments are remarkable.

To the best of our knowledge, this is the first in-vivo trial with a screening of the integrity and durability of a sealant at different time points with a consideration of oral hygiene as a co-factor. While in-vivo research offers a more realistic picture of the study subject, standardization is more difficult to achieve in comparison to using an in-vitro setup, where standardization is easy, but applicability and generalisability of results are rather poor. This study has a limitation in that there was no standardization of the brushing regimes other than providing standardized brushing instructions and handing out identical tooth brushes. There was no left-/right handed distinction. Also, oral hygiene was screened before placement of brackets, and then again 14 days following incorporation of brackets. That is, subjects were allocated to the groups of adequate or inadequate oral hygiene based on these two assessments, not considering potential improvement or deterioration afterwards.

The results of this study provide some evidence on the abatement characteristics of the longevity of a typical sealant used in orthodontics, and they indicate that a renewal of the OpalSeal layer after an average elapse of 3.5 months may be beneficial or necessary for an enduring suppression of WSL formation and frequencies. Previous studies on WSL formation following sealant application may be revisited in terms of fixed appliances orthodontic treatment duration versus presence or absence of sealant re-application. However, in this trial on the effects of time elapse and oral hygiene measures on sealant abatement, there was no assessment of WSL formation, but of sealant preservation only. That is, no conclusion can be drawn on the basis of our data that renewal of sealants after 3.5 months does indeed result in a further reduction of WSL numbers: It may also be conceivable that sealing of the enamel surfaces during the first months in fixed appliances orthodontic treatment may suffice, as it has been reported that teeth are especially susceptible to white-spot formation during this time period. Also, the interaction of sealant renewal with potentially necessary additional etching intervals needs to be considered.

### Future research

Further research is required on the subject of the mode of OpalSeal re-application (with or without additional etching) in terms of (1) reduction of frequencies of WSL, and (2) durability and abatement of sealants re-applicated the one way, or the other: As it is known that excessive phosphoric-acid etching of enamel as required prior to sealant application may trigger WSL formation itself [15], it is e.g. not clear whether enamel surfaces should be etched again prior to re-application, or if OpalSeal application renewal should be performed without a second etching interval, or not at all.

## Conclusions

The following conclusions can be drawn from the study presented here:

- Diligent screening of sealant preservation in patients treated with fixed orthodontic appliances is a necessity.
- One single sealant application is unlikely to last throughout the entire stage of orthodontic treatment with fixed appliances.
- On average, re-application of OpalSeal can be expected to be necessary after 3.5 months (week 14) in treatment. Further clinical trials should address the question if a re-application of sealants would be beneficial in terms of reducing frequencies of WSL.
- As etching of enamel surfaces is known to potentially trigger WSL formation, future research should also clarify if re-application of sealants should include an additional etching interval for an improvement of sealant durability, or not.
